# Restrictive versus liberal transfusion thresholds in very low birth weight infants: A systematic review with meta-analysis

**DOI:** 10.1371/journal.pone.0256810

**Published:** 2021-08-30

**Authors:** Peng Wang, Xing Wang, Haidong Deng, Linjie Li, Weelic Chong, Yang Hai, Yu Zhang

**Affiliations:** 1 Affiliated Hospital & Clinical Medical College of Chengdu University, Chengdu, Sichuan, China; 2 West China Hospital, Sichuan University, Chengdu, Sichuan, China; 3 Sidney Kimmel Medical College, Thomas Jefferson University, Philadelphia, Pennsylvania, United States of America; 4 Zucker School of Medicine at Hofstra/Northwell, New York, New York, United States of America; Center of Pediatrics, GERMANY

## Abstract

**Background:**

To assess the efficacy and safety of restrictive versus liberal red blood cell transfusion thresholds in very low birth weight infants.

**Methods:**

We searched MEDLINE, EMBASE, and Cochrane database without any language restrictions. The last search was conducted in August 15, 2020. All randomized controlled trials comparing the use of restrictive versus liberal red blood cell transfusion thresholds in very low birth weight (VLBW) infants were selected. Pooled risk ratio (RR) for dichotomous variable with 95% confidence intervals were assessed by a random-effects model. The primary outcome was all-cause mortality.

**Results:**

Overall, this meta-analysis included 6 randomized controlled trials comprising 3,483 participants. Restrictive transfusion does not increase the risk of all-cause mortality (RR, 0.99; 95% CI, 0.84 to 1.17; I^2^ = 0%; high-quality evidence), and does not increase the composite outcome of death or neurodevelopmental impairment (RR, 1.01, 95% CI, 0.93–1.09; I^2^ = 7%; high-quality evidence) or other serious adverse events. Results were similar in subgroup analyses of all-cause mortality by weight of infants, gestational age, male infants, and transfusion volume.

**Conclusions:**

In very low birth weight infants, a restrictive threshold for red blood cell transfusion was not associated with increased risk of all-cause mortality, in either short term or long term.

## Introduction

Up to 90% of the preterm infants with body weight at birth of less than 1000 g receive packed red blood cell (RBC) transfusion at least once during their hospital stay [1,2]. The benefits of blood transfusion include maintaining high hemoglobin and some additional benefits [3,4]. Maintaining hemoglobin at high levels was considered helpful for improving oxygen delivery and oxygen consumption [5]. While hemoglobin at low levels would have an adverse impact on growth [6]. Additionally, some studies suggested that blood transfusions can reduce the risks of hypoxemia and apnea of prematurity compared with the infants who do not receive transfusions [7,8]. However, other research showed that RBC transfusion was associated with increased risk of adverse events and complications, including retinopathy of prematurity [9–11], bronchopulmonary dysplasia [12,13], necrotizing enterocolitis [14,15]. These complications raise concerns about the safety of this treatment method [16].

Previous randomized controlled trials (RCTs) failed to demonstrate statistically significant differences in short-term outcomes, such as 30-day or in-hospital mortality in very low birth weight (VLBW) infants managed with restrictive transfusion criteria compared to those managed according to more liberal criteria, suggesting that the restrictive criteria may reduce the need for transfusion as well as not increase related side effects [17–19]. However, some cohort studies which evaluated long-term outcomes of school-age children found that cognitive impairment and reduced brain volumes may be more common with liberal transfusion thresholds [20,21]. Moreover, it is well known that blood transfusion is an important source of iron. Iron deficiency [22] and iron overload [12] have been considered as important risk factors for neurodevelopmental impairment [23]. However, current evidences do not indicate whether different blood transfusion strategy will affect hemoglobin levels and whether it will affect neurodevelopment. Since restrictive thresholds will theoretically reduce the use of RBC transfusions without increasing the risk of short-term mortality, a 2015 guideline suggest that it is preferable to adopt a restrictive criteria for VLBW requiring RBC transfusion [24,25]. However, due to the lack of randomized trials at that time, the transfusion criteria are based more on consensus of opinion of “experts” than on scientific evidence. There is an urgent need to determine whether a restrictive transfusion strategy is effective in limiting transfusions without increasing long-term mortality and morbidity in this population.

Thus, this systematic review and meta-analysis evaluated whether restrictive transfusion was associated with higher rates of death and long-term neurodevelopmental impairment in very low birth weight infants using data from the longest available follow-up.

## Methods

### Protocol and guidance

The methods and reporting of the systematic review and meta-analysis followed Preferred Reporting Items for Systematic Reviews and Meta-analyses (PRISMA) guidelines [26]. The protocol of this study was registered in PROSPERO database (CRD42020207874).

### Data sources

One of the authors (LJ)searched Ovid MEDLINE, Ovid EMBASE, and Cochrane Central Register of Controlled Trials (CENTRAL) without any language restrictions. The last search was conducted in August 15, 2020. We also performed a recursive search of the bibliographies of these selected articles as well as published systematic reviews on this topic, to identify any additional studies. We searched trial registries on WHO International Trials Registry Platform for ongoing studies or the availability of completed studies with reported results. The details of the search strategy conducted are presented in S1 Table.

### Eligibility criteria

Inclusion Criteria: (1) Population: participants considered as VLBW (birth weight <1500 g) or extremely low birth weight (ELBW: birth weight <1000 g) infants. (2) Intervention: restrictive transfusion thresholds used throughout the infants’ hospitalization. (3) Comparison intervention: liberal transfusion thresholds used throughout the infants’ hospitalization. (4) Outcome: At least one outcome of interest had to be reported. The primary outcome was all-cause mortality and long-term neurodevelopmental impairment follow-up outcome at least 18 months. All-cause mortality was categorized into short-term and long-term. Short-term mortality was defined as in-hospital mortality or 30-day mortality. Long-term mortality was defined as follow-up period of more than 12 months. Secondary efficacy outcome was the composite outcome of death or neurodevelopmental impairment with a follow-up of at least 12 months. Secondary safety outcomes included periventricular leukomalacia, bronchopulmonary dysplasia, necrotizing enterocolitis, intestinal perforation, retinopathy of prematurity stage 3 and above, sepsis, length of hospital stay, intraventricular hemorrhage grade 3 and above and hemoglobin levels. (5) Study design: randomized controlled trials.

### Study selection

Study selection followed PRISMA guidelines. After deleting duplicates, we excluded publications that were not eligible based on titles and abstracts. Then full-text articles were reviewed and either excluded or included in the analysis based on the aforementioned criteria.

Two reviewers (HD and YH) independently completed this procedure together. Conflicts in study selection were resolved by consensus, or determined by a third independent reviewer (YZ) if necessary.

### Data collection process

Two reviewers (PW and XW) independently extracted data associated with the following items onto a standardized form: (1) study characteristics: primary author, recruitment period, year of publication, geographical location and centers where study was conducted, and duration of follow-up; (2) patient characteristics: age, sex; (3) treatment characteristics. Disagreements were resolved through discussion between the two reviewers, with arbitration by a senior review if necessary. Missing information was obtained by contacting the corresponding authors of the studies.

### Risk of bias and quality of evidence

Risk of bias for all RCTs was assessed using the Cochrane Collaboration Risk of Bias tool across seven domains: random sequence generation; allocation concealment; blinding of study participants, blinding of outcome assessment, incomplete outcome data, selective reporting and other potential sources of bias. Each domain was assessed as low, unclear or high risk of bias [27]. Two reviewers (PW and XW) independently rated the confidence in the estimates of effect for each outcome to summarize results in an evidence profile by using the Grading of Recommendations, Assessment, Development, and Evaluation (GRADE) across five domains including limitation in design, inconsistency, imprecision, indirectness, and publication bias [28]. Each domain was assessed as no risk, serious risk, or very serious risk. Evidence would be considered as high-quality if all domains were rated as no risk. Disagreements were resolved through discussion between the two reviewers, with arbitration by a senior review (YZ) if necessary.

### Data synthesis

We performed statistical analyses using RevMan (5.4.0; The Cochrane Collaboration). We reported the results obtained after pooling each individual study with random-effects models to estimate pooled mean difference (MD) for continuous variable and risk ratio (RR) for dichotomous variable, with 95% confidence intervals (CIs). Heterogeneity was assessed using with the I^2^ test, I^2^ > 50% being considered substantial and needed further investigation [29]. The small study effect (ie, a tendency of smaller studies to give higher risk estimates) was assessed by using a visual estimate of the funnel plot and the regression tests Egger’s test, Begg’s test, and Harbord’s test when 10 or more trials were pooled [30]. A two-sided p value of less than 0.05 was regarded as statistically significant.

### Subgroup analysis

We did subgroup analyses for the following variables: (1) whether including only ELBW infants (Yes or No), (2) gestational age (≥ 28 weeks or < 28 weeks), (3) male infants (≥ 50% or <50%), (4) transfusion volume (≥ 20 ml/kg or < 20 ml/kg).

### Sensitivity analyses

We conducted sensitivity analyses for the primary outcome by (1) excluding trials one at a time, (2) using fixed-effect models, (3) excluding trials with less than 500 patients.

### Trial sequential analysis

Trial sequential analysis (TSA) was used to calculate the required information size in this meta-analysis by incorporating the information size and the effect size [31]. Using this method, we can explore whether cumulative data were adequately powered to draw firm conclusion. Data analysis was conducted using TSA Viewer, version 0.9.5.10 Beta (Copenhagen Trial Unit, Centre for Clinical Intervention Research, Denmark).

We used a family-wise error rate of 5%, a power of 80% (β of 20%), and a D^2^ suggested by including trials in the meta-analysis [32,33]. We used an anticipated relative risk reductions (RRRs) or relative risk increases (RRIs) of 20%, and the pooled event rate was estimated across the included studies in the TSA.

## Results

### Study selection and study characteristics

**Fig 1:**
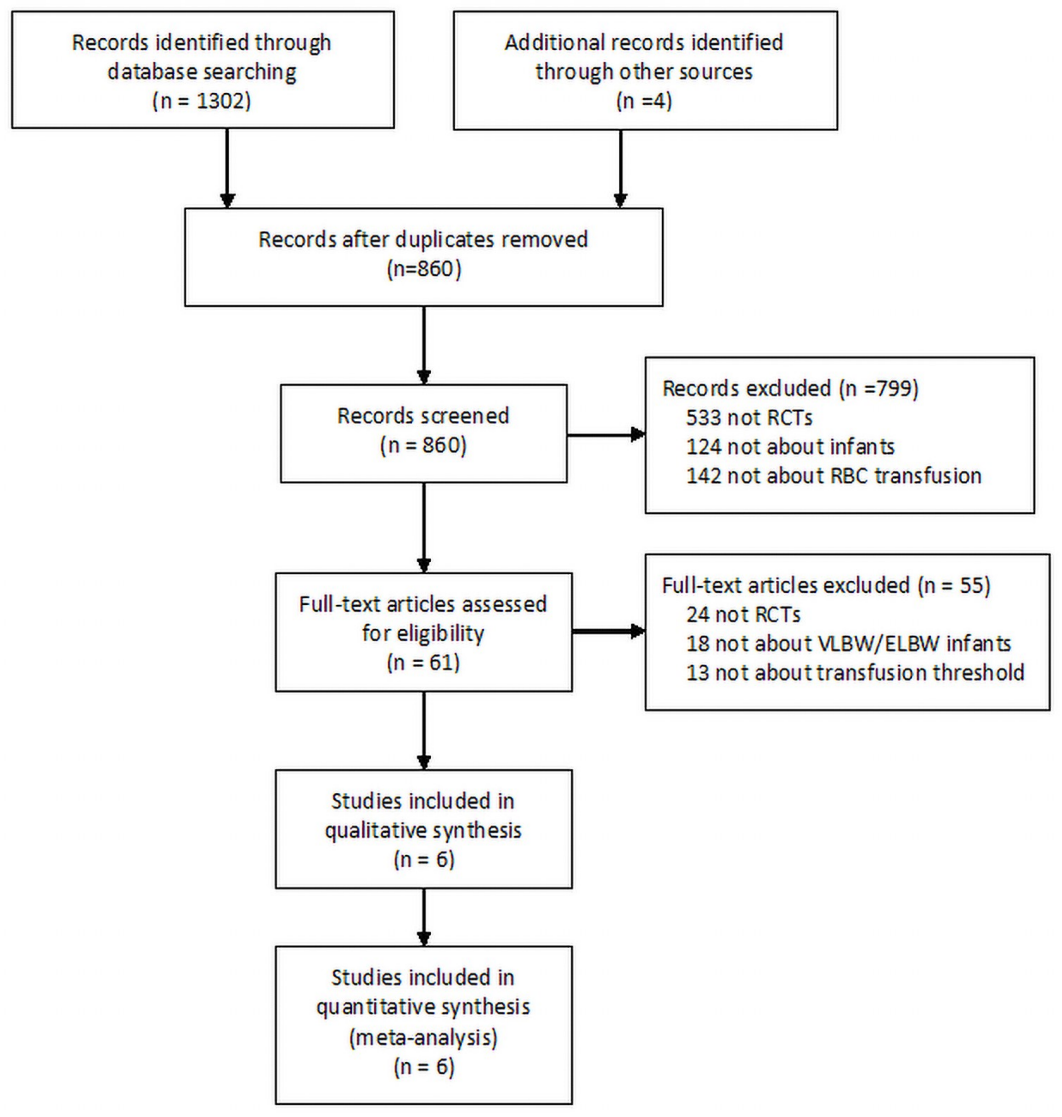
PRISMA flow chart of the studies recruited in this meta-analysis.

The initial search yielded 1303 articles. Finally, 6 trials were included in the systematic review and meta-analysis [17–19,34–37]. The PRISMA flow chart showing the publication screening process and a list of excluded studies with reasons for exclusion are provided in Fig 1.

Table 1 summarizes the study characteristics. The studies were published from 1984 to 2020. Population sizes ranged from 56 to 1,824 patients. Three trials included only extremely low birth weight infants [18,35–37]. The birth weight range that was used for inclusion are listed in Table 1. Definitions of restrictive and liberal transfusion thresholds of each trial are listed in S2 Table.

**Table 1:** Characteristics of included trials.

Trial	Setting	No. of infants	Participants ^a^	Primary outcome	Transfusion volume	Gestational age in weeks, mean (SD)	
						Restrictive	Liberal
Kirpalani 2020 [37]	41 NICUs in the United States	1824	Infants with a birth weight of less than 1000 g and a postnatal age of less than 48 hours. No. available for analysis: restrictive (n = 913), liberal (n = 911)	The primary outcome was a composite of death or neurodevelopmental impairment in infants at 22 to 26 months of age	15 ml/kg	25.9 (1.5)	25.9 (1.5)
Franz 2020 [35]	36 level III/IV neonatal intensive care units in Europe	1013	Infants with birth weights of 400 g to 999 g and a postnatal age of less than 72 hours. No. available for analysis: restrictive (n = 460), liberal (n = 491)	The primary outcome was death or disability measured at 24 months of corrected age	20 ml /kg	26.4 (1.9)	26.1 (2.0)
Chen 2009 [17]	NICU of Kaohsiung Medical University Hospital	36	Premature infants with birth weight less than 1500 g. No. available for analysis: restrictive (n = 19), liberal (n = 17)	The primary outcome was death before day 30	10 ml/kg	29.1 (3.0)	29.1 (2.7)
Kirpalani 2006 [18], Whyte 2009 [36]	10 NICUs in Canada, the United States, and Australia.	451	Infants with birth weight <1000g, and a postnatal age of less than 48 hours. No. available for analysis: restrictive (n = 223), liberal (n = 228)	For Whyte 2009, the primary outcome was a composite of death or neurodevelopmental impairment in survivors at 18 months’ corrected age. For Kirpalani 2006, the primary outcomes were mortality, ROP, BPD, and Brain injury before first neonatal discharge home	15 ml/kg	26.0 (2.0)	26.0 (2.0)
Bell 2005 [19]	University of Iowa Carver College of Medicine	103	Infants with birth weight between 500 and 1300 g. No. available for analysis: restrictive (n = 50), liberal (n = 53)	The primary outcome was mortality to discharge	15 ml/kg	27.7 (1.7)	27.8 (2.1)
Blank 1984 [34]	NICU of Lutheran General Hospital	56	Infants with birth weight <1500g. No. available for analysis: restrictive (n = 30), liberal (n = 26)	The primary outcome was length of hospital stay	——	29.4 (2.6)	29.8 (1.8)

NICU: A neonatal intensive care unit; SD: Standard deviation.

^a^ The number of participants available for the primary outcome of this meta-analysis is written.

Note that the primary outcome defined in the original article may differ from the primary outcome in this meta-analysis.

### Risk of bias and quality of evidence

Risk of bias is shown in S1 and S2 Figs. None study fulfilled all of the methodological criteria. All studies performed randomization and allocation concealment. Caregivers were not blinded in any of the studies, which some of the studies attribute to the ethical issues. Outcome assessors were blinded to the treatment group. Key findings of GRADE assessment of certainty for the main outcomes are shown in Table 2. The quality of evidence of the primary outcome was ranked as high.

**Table 2:** Summary of findings and strength of evidence of outcomes.

Outcome	No. of patients (Trials)	RR/MD (95% CI)	I^2^	Absolute effect estimates (per 1000)			Quality of the evidence
				Intervention	Control	Difference	
All-cause mortality	3325 (5)	0.99 [0.84, 1.17]	0%	140	141	-1 [-23, 24]	High
Long-term mortality	3186 (3)	0.99 [0.83, 1.17]	0%	144	145	-1 [-25, 25]	Moderate ^§^
Short-term mortality	2414 (4)	1.05 [0.86, 1.27]	0%	148	141	7 [-20, 38]	Moderate ^§^
A composite of death and neurodevelopmental impairment	3041 (3)	1.01 [0.93, 1.09]	7%	473	468	5 [-33, 42]	High
Bronchopulmonary dysplasia	3034 (5)	0.96 [0.90, 1.03]	0%	462	481	-19 [-48, 14]	Moderate ^§^
Necrotizing enterocolitis	3346 (5)	0.99 [0.84, 1.16]	0%	140	141	-1 [-23, 23]	Moderate ^§^
Retinopathy≥3	3054 (5)	0.88 [0.75, 1.03]	0%	156	177	-21 [-44, 5]	Moderate ^§^
Bowel perforation	1461 (2)	1.28 [0.75, 2.18]	45%	62	81	-17 [-16, 74]	Low ^§^^¶^
Sepsis	1494 (3)	1.06 [0.88, 1.26]	0%	226	213	13 [-26, 55]	Moderate ^§^
Length of hospital stay	3453 (6)	0.65 [-1.91, 3.21]	0%	**——**	**——**	**——**	Moderate ^§^
Intraventricular hemorrhage grade 3 or 4	1146 (3)	0.79 [0.53, 1.17]	0%	70	89	-19 [-42, 15]	Moderate ^§^
Periventricular leukomalacia	1547 (3)	0.80 [0.33, 1.93]	45%	37	46	-9 [-31, 44]	Low ^&^

^¶^ serious inconsistency.

^§^ serious imprecision.

^&^ very serious imprecision.

### All-cause mortality

**Fig 2:**
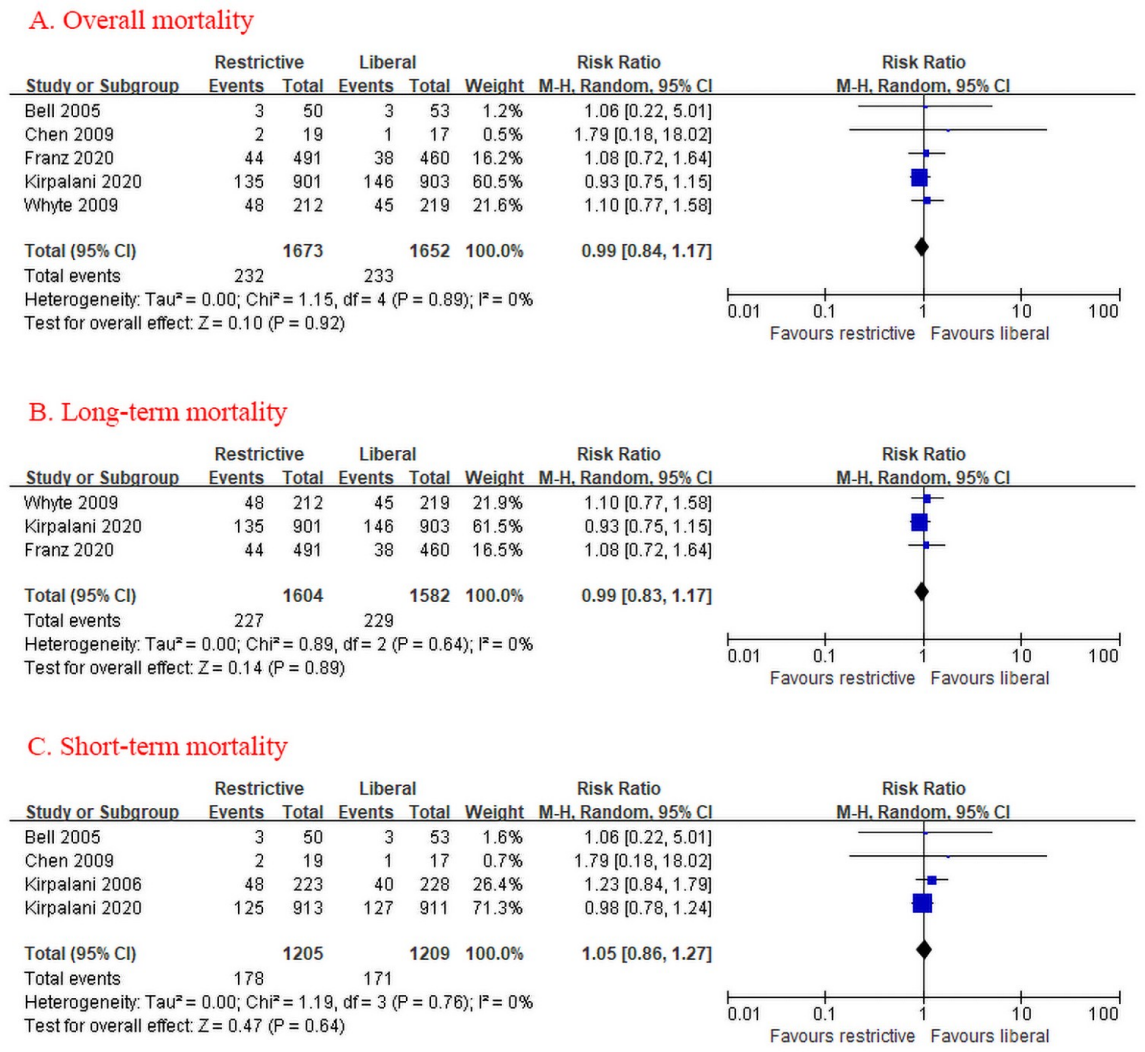
(A) Forest plot comparing overall mortality between restrictive and liberal RBC transfusion thresholds for VLBW infants. (B) Forest plot comparing long-term mortality between restrictive and liberal RBC transfusion thresholds. (C) Forest plot comparing short-term mortality between restrictive and liberal RBC transfusion thresholds. RBC: Red blood cell; VLBW: Very low birth weight.

The pooled RR showed no significant difference in overall all-cause mortality between restrictive and liberal transfusion (RR 0.99, 95% CI [0.84, 1.17]; Fig 2A). Three studies with a total of 3,186 patients investigated long-term mortality between restrictive and liberal transfusion thresholds. Overall, death occurred in 227 of 1,604 infants (14.2%) assigned to the restrictive transfusion and in 229 of 1,582 infants (14.5%) assigned to the liberal transfusion. Restrictive transfusion threshold was not associated with increased risk of long-term mortality (RR 0.99, 95% CI [0.83, 1.17]; Fig 2B). Four studies with a total of 2,414 patients investigated short-term mortality. Death occurred in 178 of 1,205 infants (14.8%) assigned to the restrictive transfusion and in 171 of 1,209 infants (14.1%) assigned to the liberal transfusion. Restrictive transfusion threshold was not associated with increased risk of short-term mortality (RR 1.05, 95% CI [0.86, 1.27]; Fig 2C). The I^2^ statistic detects no heterogeneity (I^2^ = 0%). Funnel plot analysis showed no asymmetry (S3 Fig). Since the cumulative Z-curve crossed the boundary for futility, we might accept at least a 20% RRR (S4 Fig).

**Fig 3:**
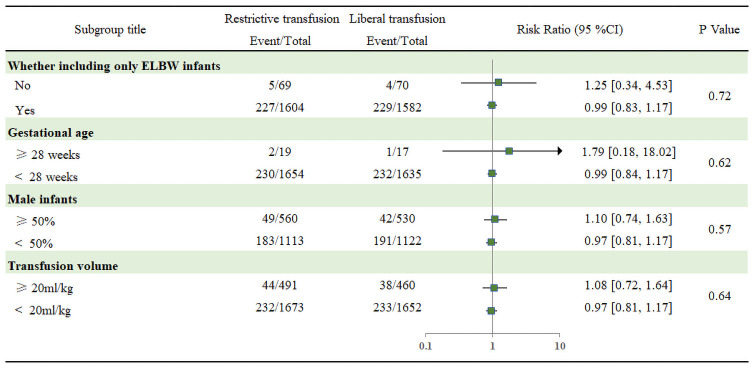
Subgroup analysis for all-cause mortality. VLBW: Very low birth weight; ELBW: Extremely low birth weight.

All-cause mortality was robust to sensitivity analyses, as all of the following were similar to the overall result: by excluding trials at each time, using fixed-effect models, and excluding trials with less than 500 patients (S3 Table). Subgroup analyses did not detect any beneficial effect within any specific subgroups on the following variables: (1) whether including only ELBW infants, (2) gestational age, (3) male infants, (4) transfusion volume (Fig 3).

### The long-term neurodevelopmental impairment

**Fig 4:**
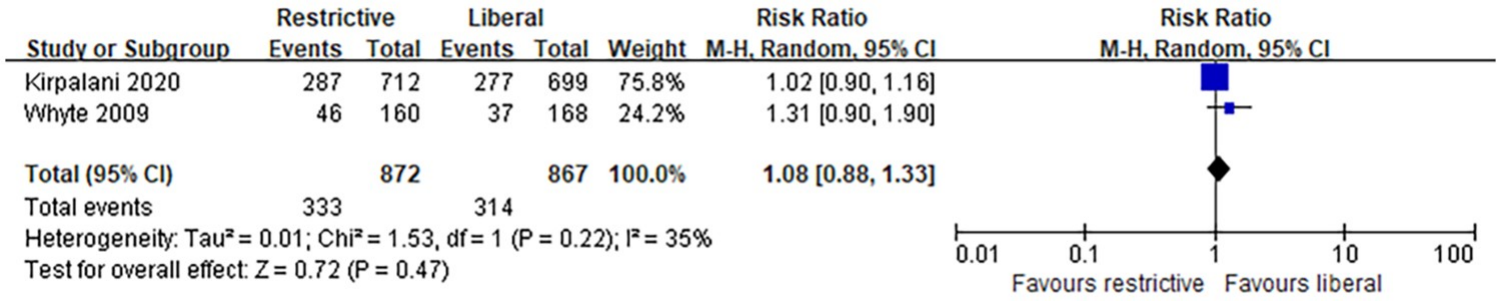


Two trials with a total of 1739 patients investigated long-term neurodevelopmental impairment between restrictive and liberal transfusion thresholds. One trial assessed the neurodevelopmental impairment within 2 years, and one trial evaluated the outcome at the 18–21 months. Fig 4 shows that no significant difference in the long-term neurodevelopmental impairment between restrictive and liberal transfusion (RR 1.08, 95% CI [0,88, 1.33]).

### Secondary efficacy outcome

**Fig 5:**
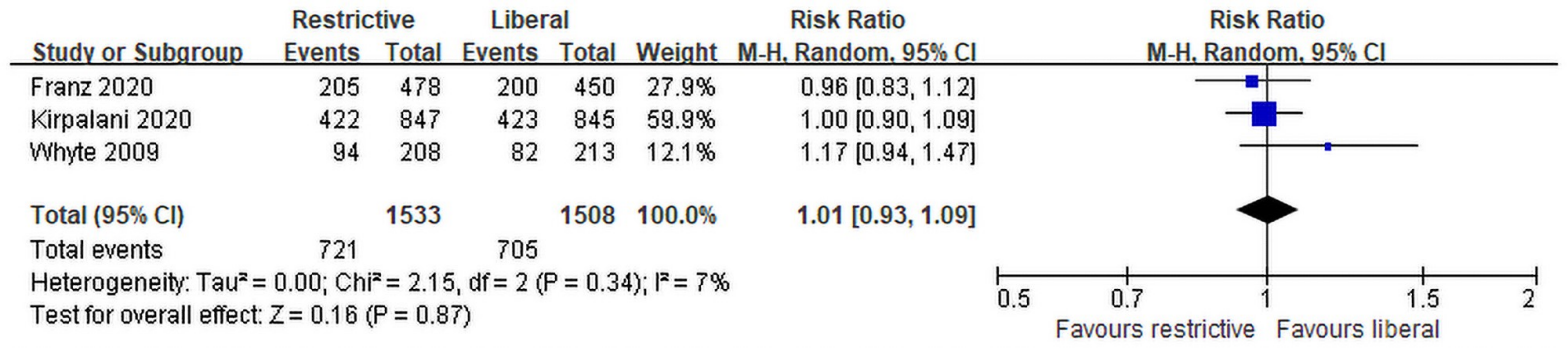
Forest plot comparing the composite death and neurodevelopmental impairment between restrictive and liberal RBC transfusion for VLBW infants. RBC: Red blood cell; VLBW: Very low birth weight.

Findings are summarized in Table 2 and Fig 5. Three trials with a combined total of 3,041 patients investigated a composite of death or neurodevelopmental impairment. A total of 721 events in restrictive transfusion and 705 events in liberal transfusion were reported. The pooled analysis showed no significant difference between restrictive and liberal transfusion thresholds (RR 1.01; 95% CI [0.93, 1.09]).

### Safety outcomes

Table 2 summarizes findings of safety outcomes. Restrictive transfusion was not associated with increased incidences of any adverse events, including bronchopulmonary dysplasia (RR 0.96; 95% CI [0.90, 1.03]), necrotizing enterocolitis (RR 0.99; 95% CI [0.84, 1.16]), retinopathy of prematurity stage 3 and above (RR 0.88; 95% CI [0.75, 1.03]), intestinal perforation (RR 1.28; 95% CI [0.75, 2.18]), sepsis (RR 1.06; 95% CI [0.88, 1.26]), length of hospital stay (RR 0.65; 95% CI [-1.91, 3.21]), intraventricular hemorrhage grade 3 and above (RR 0.75; 95% CI [0.53, 1.17]), periventricular leukomalacia (RR 0.80; 95% CI [0.33, 1.93]) and hemoglobin levels(MD -1.37; 95% CI [-2.53,-0.22]) (S5–S13 Figs).

## Discussion

To our knowledge, this study is the first comprehensive systematic review with meta-analysis to evaluate the effect of transfusion thresholds on long-term mortality in VLBW infants. In this meta-analysis of 6 trials with a total of 3,483patients, restrictive transfusion threshold was not associated with a higher rate of long-term or short-term mortality, long-term neurodevelopmental impairment, as well as the composite outcome. Also, there were no significant differences in other safety outcomes (bronchopulmonary dysplasia, necrotizing enterocolitis, retinopathy of prematurity stage 3 and above, intestinal perforation, sepsis, LOS, intraventricular hemorrhage grade 3 or 4, and periventricular leukomalacia). Subgroup analysis detected no significant findings.

### Comparison with other studies

To date, only two meta-analysis focused on the effects of restrictive versus liberal RBC transfusion thresholds on clinical outcomes in very low birth weight infants has been published [38,39]. In 2014, Ibrahim et al. [38] analyzed three trials with a total of 625 infants, concluding that restrictive RBC transfusion thresholds in VLBW infants may be utilized without incurring clinically important increases in the risk of death or major short-term neonatal morbidities. However, outcomes at extended follow-up for one of the largest studies was published in a separate paper that was missed in this meta-analysis [13]. In 2012, the other meta-analysis included 636 infants drew similar conclusions [39]. However, the previous reviews did not assess the differences of restrictive versus liberal transfusion thresholds on long-term mortality, long-term neurodevelopmental impairment, and the composite of death or neurodevelopmental impairment. Previous reviews are limited by small sample size. Recently, a meta-analysis included 18 studies revealed that RBC transfusion was associated with ROP (OR 1.50; 95% CI [1.27, 1.76]) [40]. And the use of supplemental oxygen is a risk factor to cause of ROP, but in the present meta-analysis we found that there was no difference between restrictive and liberal transfusion thresholds in retinopathy of prematurity stage 3 and above (RR 0.88; 95% CI [0.75, 1.03]). we also found that restrictive was associated with a more decrease in the hemoglobin level (MD -1.37; 95% CI [-2.53, -0.22]). Previous studies have also shown that iron deficiency [22,23] is an important risk factor for neurodevelopment, but the present study did not find any difference regarding the long-term neurodevelopmental impairment (RR 1.08, 95% CI [0,88, 1.33]), mortality or other clinical outcomes. These results are consistent with the fact that erythropoietin failed to improve neurodevelopmental outcomes despite the increase in the number of red blood cells and hemoglobin concentration [41].

The methodology and data of this study are different from those of previous meta-analysis. First, we included two recent trials on this topic [35,37] and an undated trial [36], a feature that accounted for 94.4% of the total number of patients included in this study, which helped reinforce the findings, decrease the heterogeneity, and improve the precision. Second, we chose death as the primary outcome of this study instead of adverse outcomes, which was usually selected by previous studies, because death is the most important outcome in VLBW infants. In addition, several adverse outcomes were reported, which provided a comprehensive perspective. Third, we identified several new findings, including no difference on the composite of death and neurodevelopmental impairment. Fourth, we identified several new subgroup analyses based on patients’ characteristics (whether including only ELBW infants, gestational age, male infants, transfusion volume). After careful examination of this meta-analysis using the GRADE approach and TSA method, the quality of evidence of the primary outcome was rated as high.

### Strengths and limitations

The major strength in our review is the strict methodology implemented, which followed the recommendations of the Cochrane Collaboration and PRISMA statement, including a protocol, an up-to-date literature search and study selection, data extraction, and risk of bias assessment by two independent investigators. We followed the GRADE approach to assess the degree of certainty in pooled estimates of effect and presented absolute and relative risks. Trial sequential analysis was also used for the primary outcome to explore whether cumulative data were adequately powered to evaluate outcome. This meta-analysis was larger than previous studies aimed at the same subject, and was robust despite sensitivity analyses.

Several limitations must be considered. First, there were differences across trials in inclusion criteria in terms of birth weight. However, after excluding specified trials, the results remained robust (see in S3 Table). Second, TOP trial was the largest trial we included and contributed the most to the results; nevertheless, the primary outcome remained the same after excluding it. Third, trials had different definitions of restrictive or liberal thresholds, which makes it hard to compare different trials directly head-to-head. A related problem was that there is a significant percentage of patients where the RBC transfusion was not given to protocol, or was given due to an exceptional indication such as major surgery or other emergency but not due to a hematocrit going below a threshold. The percentages between the two arms could be large enough to influence results. Since such details are only reported in the larger trials, we did not investigate this discrepancy quantitatively. Standardization of the hemoglobin or hematocrits transfusion thresholds of RBC is necessary in clinic practice. Definitions of restrictive and liberal transfusion threshold of each trial were shown in S2 Table. Fourth, we did not detect a small-study effect bias because we only included six trials. However, any potential publication bias is likely insignificant since all trials taken separately did not report any significant findings regarding mortality.

### Implications

The 2015 guidelines for transfusion therapy in neonatology declared that “the transfusion criteria used for VLBW babies are based more on consensus of opinions of “experts” than on scientific evidence” [24]. There was insufficient evidence to evaluate whether a restrictive transfusion strategy is effective in limiting transfusions without increasing morbidity and mortality in VLBW infants. Despite this, several guidelines used different thresholds to differentiate restrictive and liberal transfusion. These thresholds varied within individual countries and within individual Neonatal Units (NNUs) [5,42].

This meta-analysis suggests that in very low birth weight infants who need to receive RBC transfusions, the use of restrictive transfusion does not increase all-cause mortality,long-term neurodevelopment impairment, and the composite death and neurodevelopment impairment outcome. However, there are several differences in transfusion-related variables, including the definition of thresholds for RBC transfusion, duration of blood transfusion, and the transfusion volume. Future research is required to focus on these issues, especially to precisely define the optimal thresholds that maximize benefits and minimize harms. This new evidence should lead us to reconsider the prior recommendations.

## Supporting information

S1 Checklist PRISMA 2009 checklist. (DOCX)

S1 Fig Risk of bias summary: Review authors’ judgements about each risk of bias item for each included study. (DOCX)

S2 Fig Risk of bias graph: Review authors’ judgements about each risk of bias item presented as percentages across all included studies. (DOCX)

S3 Fig Funnel plot for all-cause mortality. (DOCX)

S4 Fig Trial sequential analysis for all-cause mortality. (DOCX)

S5 Fig Forest plot comparing RR of bronchopulmonary dysplasia between restrictive and liberal RBC transfusion for VLBW infants. (DOCX)

S6 Fig Forest plot comparing RR of necrotizing enterocolitis between restrictive and liberal RBC transfusion for VLBW infants. (DOCX)

S7 Fig Forest plot comparing RR of periventricular leukomalacia between restrictive and liberal RBC transfusion for VLBW infants. (DOCX)

S8 Fig Forest plot comparing RR of intestinal perforation between restrictive and liberal RBC transfusion for VLBW infants. (DOCX)

S9 Fig Forest plot comparing RR of retinopathy of prematurity stage 3 and above between restrictive and liberal RBC transfusion for VLBW infants. (DOCX)

S10 Fig Forest plot comparing RR of sepsis between restrictive and liberal RBC transfusion for VLBW infants. (DOCX)

S11 Fig Forest plot comparing MD of length of hospital stay between restrictive and liberal RBC transfusion for VLBW infants. (DOCX)

S12 Fig Forest plot comparing RR of intraventricular hemorrhage grade 3 and above between restrictive and liberal RBC transfusion for VLBW infants. (DOCX)

S13 Fig Forest plot comparing MD of hemoglobin levels between restrictive and liberal RBC transfusion for VLBW infants. (DOCX)

S1 Table Search strategy. (DOCX)

S2 Table Definition of restrictive and liberal transfusion threshold of each trial. (DOCX)

S3 Table Sensitivity analyses for all-cause mortality. (DOCX)
