# Supplementary material for: Restrictive versus liberal transfusion thresholds in very low birth weight infants: A systematic review with meta-analysis
Source: PLoS One. 2021 Aug 30;16(8):e0256810. doi: 10.1371/journal.pone.0256810 (PMC8405031; doi:10.1371/journal.pone.0256810)
Supplement: S4 Fig — (DOCX) [file pone.0256810.s005.docx]

**Figure S4: Trial sequential analysis for all-cause mortality.**


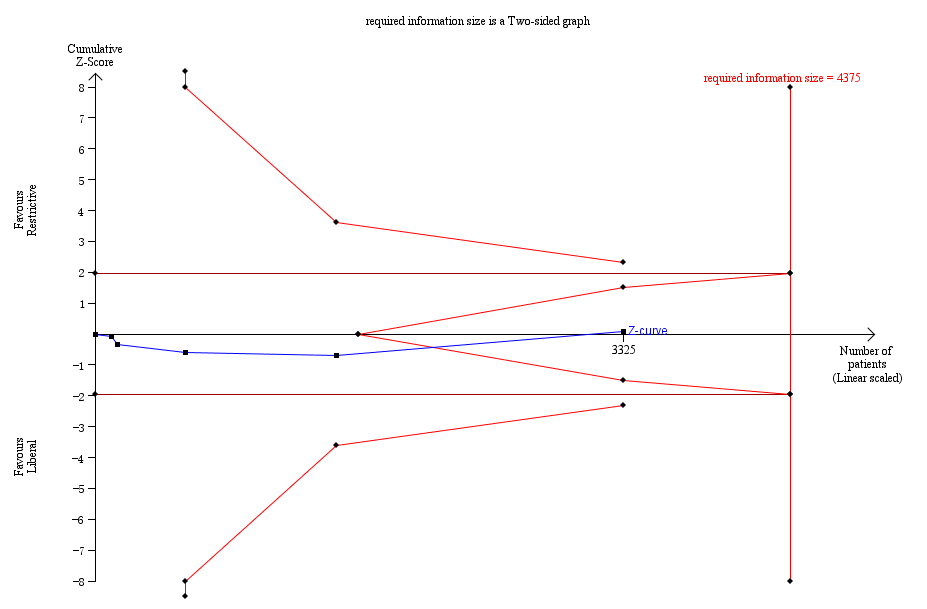


Control event proportion of 14.1%, diversity (D^2^) of 0%, alpha of 5%, power of 80% and RRR of 20%. The cumulative Z-curve crossed the boundary for futility, suggesting that we might accept at least a 20% RRR.
