# Supplementary material for: Restrictive versus liberal transfusion thresholds in very low birth weight infants: A systematic review with meta-analysis
Source: PLoS One. 2021 Aug 30;16(8):e0256810. doi: 10.1371/journal.pone.0256810 (PMC8405031; doi:10.1371/journal.pone.0256810)

**Figure S6: Forest plot comparing RR of necrotizing enterocolitis between restrictive and liberal RBC transfusion for VLBW infants.**


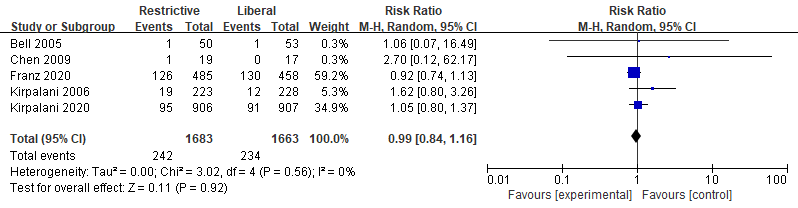

Supplement: S6 Fig — (DOCX) [file pone.0256810.s007.docx]
