# Supplementary material for: Restrictive versus liberal transfusion thresholds in very low birth weight infants: A systematic review with meta-analysis
Source: PLoS One. 2021 Aug 30;16(8):e0256810. doi: 10.1371/journal.pone.0256810 (PMC8405031; doi:10.1371/journal.pone.0256810)
Supplement: S1 Table — (DOCX) [file pone.0256810.s015.docx]

**Table S1: Search Strategy:**

| **OVID MEDLINE** | | |
| --- | --- | --- |
| 1 | exp Blood Transfusion/ | 85982 |
| 2 | exp Erythrocyte Transfusion/ | 9023 |
| 3 | 1 or 2 | 85982 |
| 4 | Blood.ab,kw,ti | 1859203 |
| 5 | ‘Transfuse*’.ab,kw,ti | 115313 |
| 6 | 4 and 5 | 79185 |
| 7 | 3 or 6 | 132981 |
| 8 | exp Infant, Low Birth Weight/ or exp Infant, Extremely Low Birth Weight/ or exp Infant/ or exp Infant, Newborn/ or exp Infant, Very Low Birth Weight/ or exp Infant, Extremely Premature/ or exp Infant, Premature/ | 1141681 |
| 9 | randomized controlled trial.pt | 512768 |
| 10 | controlled clinical trial.pt | 93834 |
| 11 | (random*or blind* or trial*).ab,kw,ti | 301337 |
| 12 | 9 or 10 or 11 | 754627 |
| 13 | exp animals/ not humans.sh | 4732465 |
| 14 | 12 not 13 | 729021 |
| 15 | 7 and 8 and 14 | 562 |
|  |  |  |
| **OVID EMBASE** | | |
| 1 | exp Blood Transfusion/ | 176629 |
| 2 | exp Erythrocyte Transfusion/ | 27585 |
| 3 | 1 or 2 | 176629 |
| 4 | Blood.ab,kw,ti | 2527296 |
| 5 | ‘Transfuse*’.ab,kw,ti | 25087 |
| 6 | 4 and 5 | 20501 |
| 7 | 3 or 6 | 183305 |
| 8 | exp Infant/ | 988588 |
| 9 | exp low birth weight/ or exp very low birth weight/ or exp extremely low birth weight/ | 62541 |
| 10 | 8 and 9 | 90953 |
| 11 | exp randomized controlled trial/ | 619947 |
| 12 | exp controlled clinical trial/ | 806522 |
| 13 | (random*or blind* or trial*). ab,kw,ti | 2578222 |
| 14 | 11 or 12 or 13 | 2781368 |
| 15 | exp animal/ | 25986665 |
| 16 | exp human/ | 21312433 |
| 17 | 15 not 16 | 4674232 |
| 18 | 14 not 17 | 2547222 |
| 19 | 7 and 10 and 18 | 360 |
|  |  |  |
| **OVID Cochrane CENTRAL** | | |
| 1 | exp Blood Transfusion/ | 3430 |
| 2 | exp Erythrocyte Transfusion/ | 567 |
| 3 | 1 or 2 | 3430 |
| 4 | Blood.ab,kw,ti | 281161 |
| 5 | Transfuse*.ab,kw,ti | 2365 |
| 6 | 4 and 5 | 2043 |
| 7 | 3 or 6 | 4883 |
| 8 | exp Infant, Extremely Low Birth Weight/ or exp Infant, Extremely Premature/ or exp Infant, Low Birth Weight/ or exp Infant, Small for Gestational Age/ or exp Infant/ or exp Infant, Newborn/ or exp Infant, Very Low Birth Weight/ | 31358 |
| 9 | 7 and 8 | 380 |
