# Supplementary material for: Restrictive versus liberal transfusion thresholds in very low birth weight infants: A systematic review with meta-analysis
Source: PLoS One. 2021 Aug 30;16(8):e0256810. doi: 10.1371/journal.pone.0256810 (PMC8405031; doi:10.1371/journal.pone.0256810)
Supplement: S2 Table — (DOCX) [file pone.0256810.s016.docx]

**Table S2: Definition of restrictive and liberal transfusion threshold of each trial.**

| Trial | Definition of restrictive and liberal transfusion thresholds |
| --- | --- |
| Kirpalani 2020 | Hemoglobin transfusion thresholds in both groups were determined according to postnatal age (highest in the first week of life, lower in each of the 2 successive weeks, and stable thereafter) and according to the use of respiratory support (a higher threshold when respiratory support was warranted). |
| Franz 2020 | In both treatment groups, the red blood cell transfusion trigger thresholds were applied from randomization to discharge home (or transfer). Trigger thresholds also depended on current state of health |
| Chen 2009 | In the “restrictive” group, the hematocrit should be maintained at more than 35% in infants with assisted ventilation, more than 30% in infants with nasal continuous positive airway pressure (CPAP) support, and more than 22% in infants breathing spontaneously. In the “liberal” group, the hematocrit should be kept at more than 45% in infants with assisted ventilation, more than 40% in infants with nasal CPAP support, and more than 30% in infants breathing spontaneously. |
| Kirpalani 2006, Whyte 2009 | The appropriate hemoglobin threshold was adjusted for capillary or arterial/venous blood sampling according to prior work. The thresholds developed were based on whether or not the infant was receiving respiratory support (assisted ventilation, continuous positive airway pressure, or supplemental oxygen) and on post-natal age. |
| Bell 2005 | While tracheally intubated for assisted ventilation (phase 1), infants in the liberal- and restrictive-transfusion groups received an RBC transfusion if their hematocrit levels fell to＜46% and＜34%, respectively. While receiving nasal continuous positive airway pressure or supplemental oxygen (phase 2), their hematocrit levels were kept at＞38% and＞28%, respectively, and if requiring neither positive pressure nor oxygen (phase 3), they were kept at＞30% and＞22%, respectively. |
| Blank 1984 | Babies in restrictive group received a transfusion if the hemoglobin level was 10.0 g/dL. The indications for transfusion in babies in liberal group were as follows: no specific hemoglobin level; preoperative surgery hemoglobin level of 10.0 g/dL or greater; tachycardia greater than 170 beats per minute for four days without primary cardiac abnormality; no weight gain for seven days with caloric intake of more than 140 calories/kg/day; and clinically notable apnea not responsive to theophylline therapy. |
