# Supplementary material for: Restrictive versus liberal transfusion thresholds in very low birth weight infants: A systematic review with meta-analysis
Source: PLoS One. 2021 Aug 30;16(8):e0256810. doi: 10.1371/journal.pone.0256810 (PMC8405031; doi:10.1371/journal.pone.0256810)
Supplement: S3 Table — (DOCX) [file pone.0256810.s017.docx]

**Table S3: Sensitivity analyses for all-cause mortality**

|  | Long-term mortality | | Short-term mortality | | Overall-mortality | |
| --- | --- | --- | --- | --- | --- | --- |
|  | RR, 95% CI | I^2^ | RR, 95% CI | I^2^ | RR, 95% CI | I^2^ |
| excluding trials with less than 500 patients | 0.96 [0.79, 1.16] | 0 | 0.98 [0.78, 1.24] | NA | 0.96 [0.79, 1.16] | 0 |
| using fixed-effect models | 0.99 [0.83, 1.17] | 0 | 1.05 [0.86, 1.27] | 0 | 0.99 [0.84, 1.17] | 0 |
| excluding trials at each time |  |  |  |  |  |  |
| Kirpalani 2020 | 1.09 [0.83, 1.44] | 0 | 1.23 [0.86, 1.76] | 0 | 1.10 [0.84, 1.44] | 0 |
| Franz 2020 | 0.97 [0.81, 1.17] | 0 | NA | NA | 0.97 [0.81, 1.17] | 0 |
| Whyte 2009 | 0.96 [0.79, 1.16] | 0 | NA | NA | 0.96 [0.80, 1.16] | 0 |
| Kirpalani 2006 | NA | NA | 0.99 [0.79, 1.24] | 0 | NA | NA |
| Bell 2005 | NA | NA | 1.05 [0.86, 1.27] | 0 | 0.99 [0.84, 1.17] | 0 |
| Chen 2009 | NA | NA | 1.04 [0.86, 1.27] | 0 | 0.99 [0.84, 1.17] | 0 |
| NA: not applicable | | | | | | |
